# Model-Based Autonomous Navigation with Moment of Inertia Estimation for Unmanned Aerial Vehicles

**DOI:** 10.3390/s19112467

**Published:** 2019-05-29

**Authors:** Hery Mwenegoha, Terry Moore, James Pinchin, Mark Jabbal

**Affiliations:** 1 Nottingham Geospatial Institute, University of Nottingham, Triumph Road, Nottingham NG7 2TU, UK; terry.moore@nottingham.ac.uk (T.M.); James.Pinchin@nottingham.ac.uk (J.P.); 2 Fluids and Thermal Engineering Research Group, University of Nottingham, Nottingham NG7 2RD, UK; Mark.Jabbal@nottingham.ac.uk

**Keywords:** GNSS, INS, VDM, Model-Based Navigation, Unscented Kalman Filter

## Abstract

The dominant navigation system for low-cost, mass-market Unmanned Aerial Vehicles (UAVs) is based on an Inertial Navigation System (INS) coupled with a Global Navigation Satellite System (GNSS). However, problems tend to arise during periods of GNSS outage where the navigation solution degrades rapidly. Therefore, this paper details a model-based integration approach for fixed wing UAVs, using the Vehicle Dynamics Model (VDM) as the main process model aided by low-cost Micro-Electro-Mechanical Systems (MEMS) inertial sensors and GNSS measurements with moment of inertia calibration using an Unscented Kalman Filter (UKF). Results show that the position error does not exceed 14.5 m in all directions after 140 s of GNSS outage. Roll and pitch errors are bounded to 0.06 degrees and the error in yaw grows slowly to 0.65 degrees after 140 s of GNSS outage. The filter is able to estimate model parameters and even the moment of inertia terms even with significant coupling between them. Pitch and yaw moment coefficient terms present significant cross coupling while roll moment terms seem to be decorrelated from all of the other terms, whilst more dynamic manoeuvres could help to improve the overall observability of the parameters.

## 1. Introduction

The dominant navigation system for low-cost, mass-market Unmanned Aerial Vehicles (UAVs) is based on Inertial Navigation System (INS)/Global Navigation Satellite System (GNSS) integration [1,2,3,4,5]. However, problems tend to arise during GNSS outages where the navigation solution error grows unboundedly [6,7,8,9]. This can happen due to intentional or unintentional corruption, even against cryptographically secured GNSS signals [10], rapid dynamics [11], loss of line of sight, and interference [12].

Rapid drift of the navigation solution during GNSS outages has been investigated extensively. Some authors have proposed using additional aiding exteroceptive sensors such as cameras [7,13,14] and range finders [1,7,15], which other than adding extra weight and cost, suffer from inherent limitations due to dependency on external sensing. Others have explored advanced integration schemes [2,3,11], and others have investigated advanced error modelling schemes [9,16], saving on weight but introducing additional software complexities.

More recently, research has been conducted on the inclusion of the Vehicle Dynamics Model (VDM) in providing a bounded navigation solution during periods of extended GNSS outages [15,17,18,19,20]. This approach preserves system autonomy while avoiding the addition of extra weight, cost, and power, essential for low-cost applications.

The use of both GNSS and Inertial Measurement Unit (IMU) measurements in aiding a VDM during extended GNSS outages with direct estimation of moment of inertia terms using an Unscented Kalman Filter (UKF) has not been explored before. Therefore, a model-based integration approach is proposed in this paper with the aforementioned architecture. The approach offers improved consistency and efficiency in the estimation of the navigation solution and model parameter terms, especially during periods of extended GNSS outage. The performance of the architecture is evaluated by a Monte Carlo simulation study with a predefined trajectory and a variable wind profile, assuming the use of commercial off-the shelf low-quality Micro-Electro-Mechanical Systems (MEMS) inertial sensors. The assessment is made in terms of navigation accuracy and filter consistency, especially during periods of extended GNSS outage.

Section 2 details current available solutions in using the VDM for navigation and the proposed architecture, focusing on similarities and differences with the available solutions. Section 3 details the performance assessment of the proposed architecture for a fixed wing UAV, highlighting the coordinate frame, equations of motion and filtering methodology. Section 4 details simulation results and the discussion of the results, and Section 5 concludes the report and highlights future work.

## 2. Solutions

A detailed description of the available solutions follows, and the proposed concept will be explained in the ensuing section, focusing on the differences and similarities with the presented solutions.

### 2.1. Available Solutions

Research explores two main concepts in using the VDM for navigation, namely, model-aided and model-based navigation. Model-aided navigation employs an INS as the main process model and a VDM as an aiding tool [17,18,19,21]. Model-based is the less common, more recent approach that uses a VDM as the main process model and an INS as the aiding system [20,22,23]. Authors have reported a similar level of accuracy with different computational efficiencies in the two approaches and some have considered multi-process model architectures, but the final solution is still derived from an INS.

**Figure 1:**
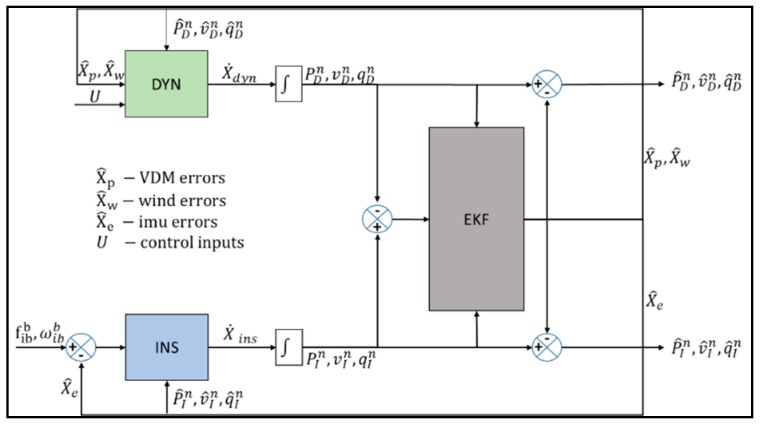
Koifman and Bar-Itzhack model-aided strapdown INS.

Koifman and Bar-Itzhack [17] presented one of the early works in using the aircraft dynamics model to aid an INS using an Extended Kalman Filter (EKF). With perfectly known dynamics, the study showed that position error for the aided INS was relatively low during the entire flight as opposed to the pure INS case. In the presence of parameter uncertainties, state vector augmentation to include model parameters was found to improve navigation performance. Further, it was indicated that, slalom manoeuvres improved observability of different modes. The integration approach considered both the aircraft dynamics model and the INS at the same level in a multi-process model as shown in Figure 1.

A multi-process model approach is inherently computationally intensive as it introduces duplicate states. Additionally, the authors indicated using low-grade inertial sensors, but the presented error stochastics suggest high-end sensors.

Julier and Hugh [24] investigated the role of vehicle process models in sensor-based navigation systems for autonomous land vehicles using an EKF. Using a high-fidelity model of a high-speed automated ground vehicle implemented in the multibody dynamics simulation ADAMS software [24], the study showed that higher-order models suffer from observability problems in VDM parameters but imposing weak constraints helps to mitigate the problem. The authors show that the error between the true vehicle and the process model manifests itself in terms of a penalty which must be applied to the process noise covariance and nature of this penalty is time varying. It was shown that modest changes to the process model reduced orientation errors by 90% and position errors by 40%. The authors focused on land vehicles using constraints in their process model which are not directly applicable to a fixed wing aircraft. However, the general principles and deductions highlight the importance of a VDM in improved navigation performance.

**Figure 2:**
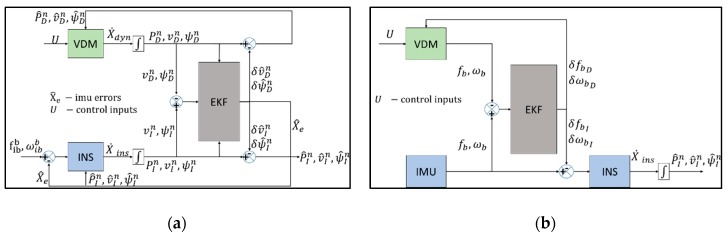
(**a**) Vehicle Dynamics Model (VDM) outputting pose estimates, (**b**) VDM outputting raw accelerations and rates.

Bryson and Sukkarieh [18] investigated the use of the VDM in aiding position, velocity, and orientation estimates provided by an INS with low-cost inertial sensors for a fixed wing UAV using an EKF. Two approaches were considered, as seen in Figure 2. The first approach compared and corrected the velocity and attitude, as predicted by both the INS and VDM. The second approach used the VDM predicted acceleration and rotation rates to provide direct calibration of the IMU. In both configurations, the INS formed the main process model and VDM aiding was activated during a GNSS outage.

With a 5% parameter uncertainty, the east position error was below 100 m for configuration 1 and above 800m for configuration 2 after 50 s of GNSS outage, indicating the superior performance of the first configuration. The good performance in configuration 1 was attributed to the marginal error growth in velocity and attitude that can be estimated and rejected with greater ease than the rapid error dynamics in acceleration and rotation rates. Both configurations did not include the direct estimation of wind and online parameter calibration and the final navigation solution was still dependent on an INS, which would be disabled in case of IMU failure.

Vissière et al. [25] reported the successful hovering flight of a model helicopter with low-cost inertial sensors by the addition of an accurate dynamics model, which improved the prediction of the EKF. Dassault Systèmes’s CATIA software was used to model 688 different parts in order to obtain the inertia matrix and centre of gravity position [25]. An autonomous outdoor flight under a 20km/h wind showed bounded position errors to within 1m vertically and 3 m horizontally. Attitude errors remained bounded to within 3 degrees in roll and pitch and 15 degrees in yaw. However, the architecture did not include a mechanism for online inertia calibration, instead CATIA was used for this purpose which can be time consuming.

Dadkhah et al. [26] investigated the use of model-aiding from knowledge of the dynamics of a helicopter to aid an Attitude Heading Reference System (AHRS) using low-cost rate gyros using an EKF. The helicopter dynamics model was developed using frequency domain system identification using attitude and position information gathered from 6 high-speed MX-40 cameras [26]. The authors argued that parametric errors in the EKF measurement stream resulting from the VDM were the main cause of suboptimal performance in gyro bias estimates. They also argued that state vector augmentation to account for correlation would improve the solution. The online calibration of model parameters was not considered even though the authors mention the potential benefits of such capability. Wind is neither estimated directly nor modelled as an external unknown in the system design in which the final navigation solution is still dependent on an INS.

**Figure 3:**
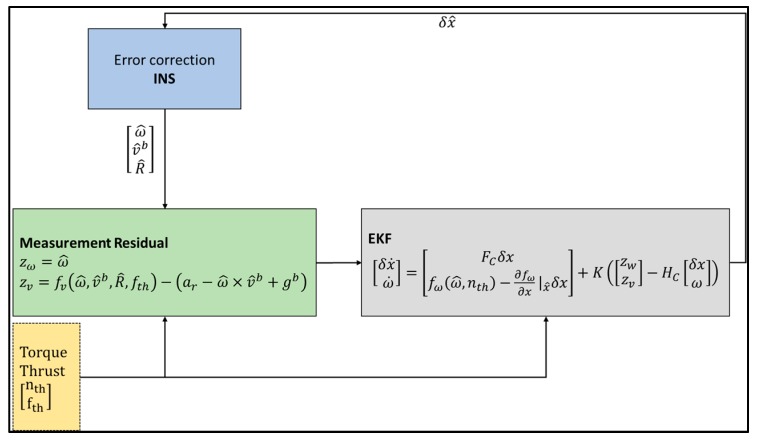
Embedded vehicle model aiding.

Vasconcelos et al. [15] implements an embedded INS with VDM for a model helicopter using low-cost inertial sensors. The approach used both error states and total states, as can be seen in Figure 3. The execution time of the embedded VDM was 400 s, 26.3% lower with both angular and linear velocity aiding, and 310 s, 42.9% lower with only linear velocity aiding, as opposed to the external VDM aiding. However, both external and embedded VDM aiding where computationally intensive as opposed to the classical INS/GNSS with the external VDM aiding execution time being 85.32% greater than the classical INS/GNSS. Both the embedded and external VDM presented similar levels of accuracy. However, the use of both total and error states presents a complex integration approach.

Crocoll et al. [19] investigates a unified INS and VDM using a modified EKF by incorporating two valid state predictions achieving a reduced state vector size and computational load over the classical VDM aiding. It was shown analytically that inclusion of position states as pseudo-measurements led to filter divergence due to zero process noise. Making the velocity and orientation states relatively equal and with a higher update rate improved the accuracy of the navigation solution. It was shown that the accuracy of the unified approach is similar to that presented in Koifman and Bar-Itzhack [17]. The ability for online parameter calibration [27] and wind estimation [28] investigated at a later stage, revealed significant navigation performance improvements. Their model, however, was for a quadrotor and ignored rotational dynamics.

Sendobry [22] completely avoids the use of duplicate states by propagating the state vector using the VDM. The state vector is augmented to include vehicle accelerations in the EKF and applied to a quadrotor. The quadrotor propulsion model was parameterised through wind tunnel experiments. An experimental investigation showed the robustness of the proposed solution using a ground vehicle. The position solution showed a drift-free navigation performance near buildings where the raw GNSS solution presented erroneous measurements and sometimes even total outage. The architecture, however, was not investigated during periods of extended GNSS outage and simulation studies were based on a quadrotor and did not consider a fixed wing aircraft.

Khaghani and Skaloud [20,29] present an extension to the model-based approach presented by Sendobry [22,30] with specific application to fixed wing UAVs. Simulation results indicated 2 orders of magnitude improvement in navigation performance from the standard INS/GNSS integration using an EKF. The approach included the estimation of wind and model parameters but did not include the online calibration of moment of inertia terms and an account of the influence of their perturbation on navigation performance was not made. In further developments, experimental results indicated attitude errors of a VDM/GNSS integration being 1 to 2 orders of magnitude larger than the conventional INS/GNSS integration [31]. This was attributed mostly to unresolved errors in the moment terms that resulted to poor attitude determination in the absence of IMU data [29].

Zahran et al. [32] investigates the use of hybrid machine learning to train a VDM and enhance inertial navigation accuracy during periods of GNSS outage using low-cost inertial sensors in a quadcopter. The machine learning module (regression and classification) acts as a substitute, providing a position and velocity solution during periods of GNSS outage. An EKF with 21 states is used as the fusion filter with regression and classification schemes resulting in lower position errors during GNSS outages, as opposed to using a regression only scheme. The model, however, did not include a mechanism for online inertia tensor estimation during a GNSS outage. Further, training of the VDM happens only during periods of GNSS availability.

Mohammadkarimi and Nobahari [33], investigated a model-aided inertial navigation approach during landing of a UAV. The algorithm estimates and removes the Ground Effect uncertainties during the last phase of landing in close proximity to the ground. The architecture utilises both a Kalman filter (KF) and an UKF for INS state propagation and VDM state propagation, respectively. With perturbations in model parameters the integration architecture is seen not to handle estimation of a variable wind profile without an air data system. Moreover, the model included duplicate states and did not include a mechanism for online inertia estimation.

### 2.2. Proposed Concept

As mentioned earlier, a model-based integration approach is proposed that includes the estimation of wind, IMU errors, model parameters and moment of inertia terms. This is achieved using direct IMU and GNSS measurements. The key concept with a model-based approach is to use available control inputs from the autopilot system to drive the navigation solution using the dynamics model of the aircraft. A feature unique to the proposed architecture is the online estimation of moment of inertia terms without additional sensors and hardware setup in a fixed wing UAV. This improves not only the confidence in the navigation solution but also allows to meet stringent size, weight, and power requirements in low-cost applications. The architecture is platform-specific and therefore requires careful modelling.

The proposed architecture is preferred over the model-aided architectures [18,26,32] for a number of reasons. In low-cost applications, the quality of the inertial sensors used is relatively low and inherently affected by significant noise sources. In case of a GNSS outage, the noise in these sensors will cause rapid drift in the navigation solution in a short time and, in case of an IMU failure, the navigation solution is disabled altogether. Further, the use of direct IMU measurements to drive the navigation solution can become unreliable in the presence of significant thermal loading unless thermal models [16] are used to eliminate stochastic variations with temperature. Accurate IMU error modelling requires considerable time and effort and, in some cases, special equipment. This is avoided in the proposed architecture since the VDM is unaffected by thermal loading and uses the IMU and GNSS measurements only to update the navigation solution. Moreover, INS-dependent solutions are generally affected by secondary effects such as coning and sculling [12,15,25] as a result of vibrations on the host platform which, if not compensated, can cause significant drift in the navigation solution. The VDM-derived solution is unaffected by the platform’s vibrations making the architecture considerably robust. Multi-process models [17], in which the final navigation solution depends on the INS, have similar limitations and therefore the proposed approach is preferred over them as well.

The proposed approach is also preferred over the more recent model-based schemes [20,22] even though similarities exist in using the VDM as the main process model. Full-scale oscillation tests or other related inertia modelling schemes take considerable time and require special equipment. The proposed concept does not rely on an accurate inertia matrix allowing the estimation of the terms in flight. This minimizes the effort in system design and improves confidence in the derived navigation solution, especially in the presence of perturbations. Moreover, the process model adopted allows for the variation of these terms during the course of a flight and even though not investigated in this work, could improve the robustness of a model-based approach in applications where mass and moment of inertia could change allowing for a more general application of the scheme.

## 3. Performance Assessment

This section details the performance assessment of a model-based navigation architecture with the VDM as the main process model, aided by the IMU and GNSS measurements using a UKF. A description of the coordinate frame, atmospheric model, equations of motion and filtering methodology follows.

### 3.1. Coordinate Frame

**Figure 4:**
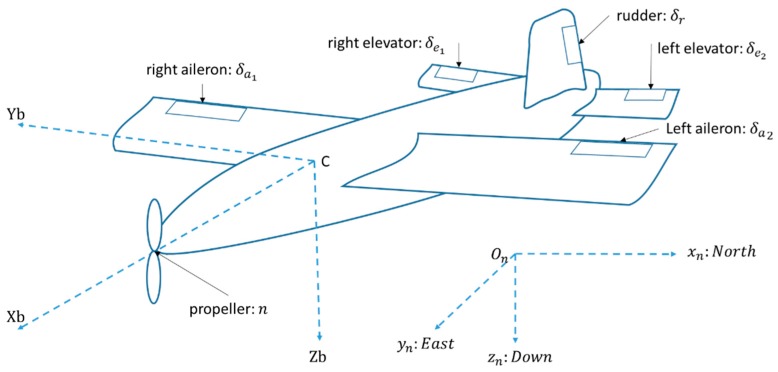
Aircraft configuration.

A local navigation frame initialised at the position of the airplane’s centre of gravity just before take-off is treated as an inertial frame. The body-fixed frame defines a right-handed orthogonal coordinate frame with the origin at the centre of gravity of the aircraft, as can be seen in Figure 4.

Inertial speed in the body frame ($v^{b}$) is given as the sum of wind speed in the local navigation frame ($W^{n}$) transformed to the body frame and airspeed vector in the body frame ($V^{b}$).

(1) $$v^{b}{\;=\;V}^{b}{+\;R}_{n}^{b}W^{n}$$

where ‘$R_{n}^{b}$’ is the rotation matrix from the local level frame to the body frame.

The airspeed, angle of attack (α), sideslip angle (β), and dynamic pressure ($\bar{q}$ ) are represented as:

(2) $$V^{b}=\sqrt{u_{T}^{2}\;+\;v_{T}^{2}+\;w_{T}^{2}}\;,\;\alpha \;=\;\arctan\left(\frac{w_{T}}{u_{T}}\right),\;\beta \;=\;\arcsin\left(\frac{v_{T}}{V^{b}}\right),\;\bar{q\;}=\;\frac{{\rho V}^{b^{2}}}{2}$$

where, ‘$u_{T}$’, ‘$v_{T}$’ and ‘$w_{T}$’ represent the components of the airspeed vector in the body frame.

### 3.2. Atmospheric Model

The atmospheric model adopted for use is modelled in accordance with the international standard atmosphere [34,35] given by:

(3) $${T\;=\;T}_{0}{[1\;+\;\mathrm{ah}/T}_{0}]\;\rho \;=\;\frac{p_{o}{\left[{1\;+\;\mathrm{ah}/T}_{0}\right]}^{5.2561}}{\mathrm{RT}}\;$$

where ‘ρ’ is the density, ‘R’ is the specific gas constant, ‘To’ is the sea level temperature, ‘$p_{o}$’ is the sea level pressure, and ‘a’ is the lapse rate [34]. This atmospheric model is valid up to an altitude of 11,000 m and therefore can be used to capture the relatively small altitude variations considered in simulation.

### 3.3. Equations of Rigid-Body Motion

For the purpose of simulation, it is assumed that the aircraft is flying over a small region of the earth and thus the earth is assumed locally flat ($R_{\mathrm{earth}}\to \infty$) and therefore, centripetal acceleration resulting from earth’s curvature is neglected. Coriolis acceleration due to rotation of the earth is neglected and thus the earth is treated as an inertial (Galilean) frame of reference, where Newton’s laws are applicable. The equations of motion are presented as:

(4) $$\left[\begin{array}{c} \dot{x_{N}}\;\\ \dot{x_{E}}\;\\ \dot{x_{D}} \end{array}\right]{=\;R}_{b}^{n}\left[\begin{array}{c} v_{x}^{b}\;\\ v_{y}^{b}\\ {\;v}_{z}^{b} \end{array}\right],\;R_{b\;}^{n}=\;\left[\begin{array}{ccc} \cos\psi \cos\theta & \;\;\;\cos\psi \sin\phi \sin\theta -\cos\phi \sin\psi & \;\;\;\sin\phi \sin\psi +\cos\phi \cos\psi \sin\theta \\ \cos\theta \sin\psi & \;\;\;\cos\phi \cos\psi +\sin\phi \sin\psi \sin\theta & \;\;\;\cos\phi \sin\psi \sin\theta -\cos\psi \sin\phi \\ -\sin\theta & \cos\theta \sin\phi & \cos\phi \cos\theta \end{array}\right]$$

where ‘$x_{N}{,\;x}_{E}{,\;x}_{D}$’ represent position in the North, East, and Down direction, ‘$R_{b}^{n}$’is the rotation matrix from the body frame to the local level frame, and ‘ϕ, θ, ψ’ represent the Euler angles: roll, pitch, and yaw, respectively.

(5) $$\left[\begin{array}{c} \dot{v_{x}^{b}}\;\\ \dot{v_{y}^{b}}\\ \;\dot{v_{z}^{b}} \end{array}\right]=\left[\begin{array}{c} -\mathrm{gsin}\theta \\ \;\mathrm{gsin}\phi \cos\theta \\ \;\mathrm{gcos}\phi \cos\theta \end{array}\right]+\frac{1}{m}\left[\left(\begin{array}{c} F_{T}\\ 0\\ 0 \end{array}\right){+R}_{w}^{b}\left(\begin{array}{c} F_{X}^{w}\\ F_{Y}^{w}\\ F_{Z}^{w} \end{array}\right)\right]-\left[\begin{array}{c} \omega_{y}v_{z\;}^{b}{-\;\omega }_{z}v_{y}^{b}\\ \omega_{z}v_{x}^{b}{\;-\;\omega }_{x}v_{z}^{b}\\ \omega_{x}v_{y}^{b}{\;-\;\omega }_{y}v_{x}^{b} \end{array}\right]\;R_{w}^{b}\;=\;\left[\begin{array}{ccc} \cos\alpha \cos\beta & \;\;\;-\cos\alpha \sin\beta & \;\;\;-\sin\alpha \\ \;\;\;\sin\beta & \;\;\;\cos\beta & \;\;\;0\\ \cos\beta \sin\alpha & \;\;\;-\sin\alpha \sin\beta & \;\;\;\cos\alpha \end{array}\right]$$

where ‘m’ is the mass and ‘$R_{w}^{b}$’ is the rotation matrix from the wind frame to the body frame. ‘$F_{T}$’ is the thrust force along the body *x*-axis, ‘$F_{Z}^{w}$’ is the lift force in the wind frame, ‘$F_{Y}^{w}$’ is the lateral force, and ‘$F_{X}^{w}$’ is the drag force in the wind frame and ‘g’ is the acceleration due to gravity.

(6) $$\left[\;\begin{array}{c} \dot{\phi }\\ \dot{\theta }\\ \dot{\psi } \end{array}\right]=R_{\phi }\left[\;\begin{array}{c} \omega_{x}\\ \omega_{y}\\ \omega_{z} \end{array}\right],\;R_{\phi }\;=\;\left[\begin{array}{ccc} 1 & \tan\theta \sin\phi \; & \tan\theta \cos\phi \\ 0 & \cos\phi & -\sin\phi \\ 0 & \sin\phi /\cos\theta & \cos\phi /\cos\theta \; \end{array}\right]$$

where ‘$\omega_{x}{,\;\omega }_{y}{,\;\omega }_{z}$’ represent the roll, pitch, and yaw angular rates around the respective body axes.

(7) $$\left[\;\begin{array}{c} \dot{\omega_{x}}\\ \dot{\omega_{y}}\\ \dot{\omega_{z}} \end{array}\right]\;=\;{\left(I^{b}\right)}^{-1}\left(\left[\begin{array}{c} M_{x}^{b}\\ {\;M}_{y}^{b}\;\\ M_{z}^{b} \end{array}\right]-\left[\begin{array}{c} \omega_{x}\\ \omega_{y}\\ \omega_{z} \end{array}\right]{\times \;I}^{b}\left[\begin{array}{c} \omega_{x}\\ \omega_{y}\\ \omega_{z} \end{array}\right]\right),\;I^{b}\;=\;\left[\begin{array}{ccc} I_{\mathrm{xx}} & 0 & I_{\mathrm{xz}}\\ 0 & I_{\mathrm{yy}} & 0\\ I_{\mathrm{zx}} & 0 & I_{\mathrm{zz}} \end{array}\right]\;$$

where ‘$M_{x}^{b}{,\;M}_{y}^{b}{,\;M}_{z}^{b}\;$’ represent the roll, pitch, and yaw moments around the body axes respectively, and ‘$I^{b}$’ represents the body-fixed inertia matrix with ‘$I_{\mathrm{xx}}{,\;I}_{\mathrm{yy}}{,\;I}_{\mathrm{zz}}{,\;I}_{\mathrm{xz}}$’ representing the components of the inertia matrix about the respective axes.

The propeller first-order dynamics model is given by:

(8) $$\dot{n}=\;-\frac{1}{\tau_{n}}n\;+\;\frac{1}{\tau_{n}}n_{c}$$

where ‘$n_{c}$’ represents the commanded propeller speed and ‘$\tau_{n}$’ is the time constant. Other actuators could also be modelled with similar dynamics but for simplicity this is not considered.

In developing the equations of motion, mass (m) and moments of inertia ($I^{b}$) are assumed constant. The aerodynamic forces ($F_{X}^{w}{,F}_{Y}^{w}{,\;F}_{Z}^{w}$) and thrust force ($F_{T}$) are represented as:

(9) $$F_{Z}^{w}=\bar{q}S({\mathrm{CF}}_{Z1}+{\mathrm{CF}}_{Z_{\alpha }}\alpha )$$

(10) $$F_{Y}^{w}=\bar{q}S{\mathrm{CF}}_{Y1}\beta$$

(11) $$F_{X}^{w}=\bar{q}S({\mathrm{CF}}_{X1}+{\mathrm{CF}}_{X_{\alpha }}\alpha +{\mathrm{CF}}_{X_{\alpha 2}}\alpha^{2}+{\mathrm{CF}}_{X_{\beta 2}}\beta^{2})$$

(12) $$F_{T}{\;=\;\rho n}^{2}D^{4}{(\mathrm{CF}}_{T_{1}}{+\mathrm{CF}}_{T_{2}}{J\;+\;\mathrm{CF}}_{T_{3}}J^{2}\;)$$

where, $J\;=\;\frac{V}{D\pi n}$ and ‘n’ is the propeller speed, ‘${\mathrm{CF}}_{{\left[X\;Y\;Z\right]}_{j}}$’ are the aerodynamic force derivatives and ‘${\mathrm{CF}}_{T_{i=[1\;2\;3]}}$’ are the thrust derivatives, ‘S’ is the wing area, and ‘D’ is the propeller diameter.

The aerodynamic moments are represented as:

(13) $$M_{X}^{b}=\bar{q}\mathrm{Sb}({\mathrm{CM}}_{X_{\delta_{\alpha }}}\delta_{\alpha }+{\mathrm{CM}}_{X_{{\bar{\omega }}_{x}}}{\bar{\omega }}_{x}+{\mathrm{CM}}_{X_{{\bar{\omega }}_{z}}}{\bar{\omega }}_{z}+{\mathrm{CM}}_{X_{\beta }}\beta )\;{\bar{\omega }}_{x}=\frac{\omega_{x}b}{2V},\;{\bar{\omega }}_{z}=\frac{\omega_{z}b}{2V}$$

(14) $$M_{Y}^{b}=\bar{q}S\bar{c}({\mathrm{CM}}_{Y_{\delta_{e}}}\delta_{e}+{\mathrm{CM}}_{Y_{{\bar{\omega }}_{y}}}{\bar{\omega }}_{y}+{\mathrm{CM}}_{Y_{\alpha }}\alpha +{\mathrm{CM}}_{Y_{1}})\;{\bar{\omega }}_{y}=\frac{\omega_{y}\bar{c}}{2V}$$

(15) $$M_{Z}^{b}=\bar{q}\mathrm{Sb}({\mathrm{CM}}_{Z_{\delta_{r}}}\delta_{r}+{\mathrm{CM}}_{Z_{{\bar{\omega }}_{z}}}{\bar{\omega }}_{z}+{\mathrm{CM}}_{Z_{\beta }}\beta )$$

where, ‘c¯’ is the wing cord, ‘b’ the wing span, ‘${\mathrm{CM}}_{{\left[X\;Y\;Z\right]}_{j}}$’ are the moment derivatives, and ‘$\delta_{\alpha },\;\delta_{e},\;\delta_{r}$’ are the aileron, elevator, and rudder deflections, respectively.

### 3.4. Filtering Methodology

An unscented Kalman filter [4,36,37] is used to provide an integrated navigation solution where the VDM forms the main process model and the inertial sensors and GNSS provide correcting measurements. As mentioned earlier, the state vector is augmented to include the vehicle navigation states ($X_{n}$), IMU errors ($X_{e}$), wind velocity ($X_{w}$), and 22 VDM parameters and 4 moment of Inertia terms ($X_{P}$). A UKF captures higher-order moments and avoids biases introduced from first-order linearization assumptions. The UKF avoids the derivation of the Jacobian matrices, a nontrivial aspect of a highly nonlinear system. A complete description of the UKF is not given here but the interested reader can see the referenced text.

#### 3.4.1. Process models

The VDM states ($X_{n}$) are propagated using the equations of motion already presented directly, without the need for any linearization.

IMU errors in the navigation filter are modelled as a random constant superposed in a random walk process given by:

(16) $${\dot{X}}_{e}=G_{\mathrm{ee}}W_{e}\;X_{e}\;=\;{\left[b_{\mathrm{ax}}{\;b}_{\mathrm{ay}}{\;b}_{\mathrm{az}}{\;b}_{\mathrm{gx}}{\;b}_{\mathrm{gy}}{\;b}_{\mathrm{gz}}\right]}^{T}\;b_{a|g[x\;y\;z]}\;:\mathrm{Accelerometer}\;\mathrm{and}\;\mathrm{Gyroscope}\;\mathrm{bias}$$

where, ‘$G_{\mathrm{ee}}$’ represents the noise shaping matrix and ‘$W_{e}$’ is the noise vector. The actual sensor model implementation follows a first-order Gauss–Markov (GM) process. Bias variations with temperature, quantization noise, and scale factors are not considered in the simulation.

(17) $${\dot{X}}_{e}=-\beta_{e}X_{e}+n_{X_{e}}$$

where, ‘$n_{\mathrm{Xe}}$’ represents the GM process driving noise and ‘$\beta_{e}$’ is the inverse of the correlation time.

Wind velocity in the navigation filter is modelled as a random walk process to capture small transitions.

(18) $${\dot{X}}_{w}=G_{w}W_{w}\;X_{w}\;=\;{\left[w_{N}{\;w}_{E}{\;w}_{D}\right]}^{T}$$

where, ‘$G_{\mathrm{ww}}$’ represents the wind vector noise shaping matrix and ‘$W_{w}$’ is the noise vector. The actual wind implementation in simulation follows a first order Gauss–Markov process with a constant component having a magnitude of 3.8 m/s, a correlation time of 200s, and process uncertainty of 0.1m/s.

Most of the VDM parameters and inertia terms are static (${\dot{X}}_{p}=0$) otherwise changes in mass distribution during flight could change the mass moment of inertia terms and therefore, for generality, the parameters are modelled as a random walk process with very small process noise to capture small transitions in time. It should be noted that during the simulation the parameters are fixed.

$$\mathrm{Xp}\;=\;[\begin{array}{c} {\mathrm{CF}}_{T_{1}}{\;\mathrm{CF}}_{T_{2}}{\;\mathrm{CF}}_{T_{3}}{\;\mathrm{CF}}_{X_{1}}{\;\mathrm{CF}}_{X_{\alpha }}{\;\mathrm{CF}}_{X_{\alpha 2}\;}{\mathrm{CF}}_{X_{\beta 2}}{\;\mathrm{CF}}_{Z_{1}}\;\\ {\mathrm{CF}}_{Z_{\alpha }}{\;\mathrm{CF}}_{Y_{1}}{\;\mathrm{CM}}_{X_{\delta_{\alpha }}}{\;\mathrm{CM}}_{X_{\beta }}{\;\mathrm{CM}}_{X_{{\bar{\omega }}_{x}}}{\;\mathrm{CM}}_{X_{{\bar{\omega }}_{z}}}\;{\mathrm{CM}}_{Y_{1}}\;\\ {\mathrm{CM}}_{Y_{\alpha }}{\;\mathrm{CM}}_{Y_{\delta_{e}}}{\;\mathrm{CM}}_{Y_{{\bar{\omega }}_{y}}}{\;\mathrm{CM}}_{Z_{\delta_{r}}}{\;\mathrm{CM}}_{Z_{{\bar{\omega }}_{z}}}\;{\mathrm{CM}}_{Z_{\beta }}\;\tau_{n}\\ I_{\mathrm{XX}}{\;I}_{\mathrm{YY}}{\;I}_{\mathrm{ZZ}}{\;I}_{\mathrm{XZ}} \end{array}]$$

#### 3.4.2. Observation Model

The measurement vector consists of IMU ($Z_{\mathrm{imu}}$) and GNSS receiver ($Z_{\mathrm{gnss}}$) measurements presented as:

(19) $$Z\;=\;{\left[Z_{\mathrm{imu}}{\;Z}_{\mathrm{gnss}}\right]}^{T}$$

The discrete measurement model ($z_{k}$) is a function of the measurement function (h) presented as:

(20) $$z_{k}\;=\;h\left[x_{k}{,\;u}_{k}\right]{+r}_{k}$$

where ‘$x_{k}$’ is the predicted state vector at the current time index ‘k’, ‘$u_{k}$’ is the known control input vector at the current time index and, ‘$r_{k}$’ is the white sequence vector. The expectation operator on the white sequence and its transpose is given by:$\;E\left[r_{k}r_{k}^{T}\right]{\;=\;R}_{k}$. ‘$R_{k}$’ is the measurement covariance matrix.

The measurement function for the IMU ($h_{\mathrm{imu}}$) is presented as:

(21) $$h_{\mathrm{imu}\;}=\;\left[\begin{array}{c} h_{\mathrm{accel}}\\ h_{\mathrm{gyro}} \end{array}\right]\;=[\begin{array}{c} \dot{v}-g+[\omega \times ]v_{\left[3\times 1\right]}+\;X_{e}\left(\left[1\;2\;3\right]\right)\\ \omega_{\left[3\times 1\right]\;}{+\;X}_{e}\left(\left[4\;5\;6\right]\right) \end{array}]\;\left[\omega \times \right]\;=\;\left[\begin{array}{ccc} 0 & {-\omega }_{z} & \omega_{y}\\ \omega_{z} & 0 & {-\omega }_{x}\\ {-\omega }_{y} & \omega_{x} & 0 \end{array}\right]\;$$

where $\omega_{\left[3\;\times \;1\right]}\;=\;{\left[\omega_{x}{\;\omega }_{y}{\;\omega }_{z}\right]}^{T}$ and $v_{\left[3\;\times \;1\right]}\;=\;{\left[v_{x}^{b}{\;v}_{y}^{b}{\;v}_{z}^{b}\right]}^{T}$. The measurement function for the GNSS receiver ($h_{\mathrm{gnss}}$) is presented as:

(22) $$h_{\mathrm{gnss}}\;=\;\left[\begin{array}{c} x_{N}\\ x_{E}\\ x_{D} \end{array}\right]$$

#### 3.4.3. Structure

**Figure 5:**
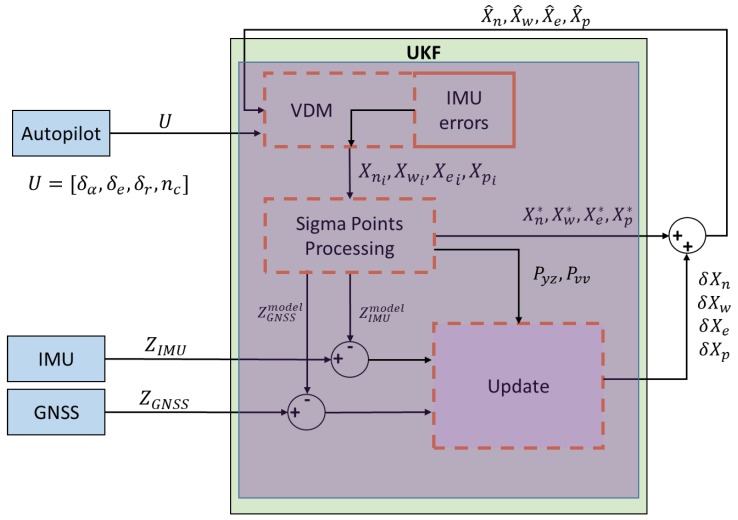
VDM Unscented Kalman Filter (UKF) Structure as implemented in simulation. ‘${\delta X}_{n}\;,{\;\delta X}_{w},\;{\delta X}_{e},\;{\delta X}_{p}$’ are estimated errors in the navigation, wind, Inertial Measurement Unit (IMU) bias states and model parameters, respectively. ‘$X_{\left[n\;w\;e\;p\right]i}$’ represents the generated sigma points for the navigation, wind, IMU bias states and model parameters respectively; ‘$X_{\left[n\;w\;e\;p\right]}^{*}$’ represents the corresponding weighted averages of the propagated sigma points; ‘${\hat{X}}_{\left[n\;w\;e\;p\right]}$’ represents the updated state vector using the true and predicted measurements ($Z_{\left[\mathrm{IMU}\;\mathrm{GNSS}\right]}^{\mathrm{model}}$ ); ‘$P_{\mathrm{yz}}$’ and ‘$P_{\mathrm{vv}}$’ represent the cross-covariance and innovation covariance, respectively.

The process model and observation model together are used in the implementation of the UKF, as can be seen in Figure 5. It should be noted that the states are propagated internally in the sigma points processing block.

**Figure 6:**
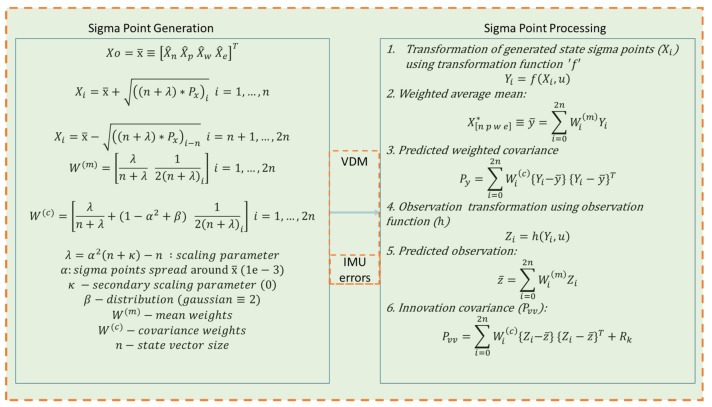
VDM UKF Sigma Points Processing Block. The sigma point processing block uses the mean and covariance weights to estimate the a priori states and the resulting covariance matrices.

The sigma points processing block has been expanded, as can be seen in Figure 6.

The update block outputs correction values for the states and consists of standard Kalman filter update routines after the mean and covariance have been estimated in the sigma point processing block. The interested user can refer to Gelb et al. [38] and Brown and Hwang [4] for a complete overview.

#### 3.4.4. Implementation

Table 1 presents a summary of the error characteristics implemented in simulation. The filter does not use the true error stochastics to maintain a situation close to reality that error stochastics cannot be precisely known but only approximated. Since the application is targeting low-cost applications, most of these stochastics, in an experimental implementation, would come either directly from Manufacturer specifications or characterisation techniques such as Allan Variance or Generalised Wavelet Moments.

For the GNSS receiver, independent white noise on each channel (north, east, down) is modelled with a standard deviation of 1 m and the sampling frequency is 1 Hz.

The initial uncertainties considered for the augmented state vector are presented in Table 2.

The process noise for the filter is presented in Table 3 in terms of the standard deviations.

For a full list of aerodynamic coefficient, moments of inertia and their values considered for the purpose of simulation, the reader is directed to Ducard [35].

**Figure 7:**
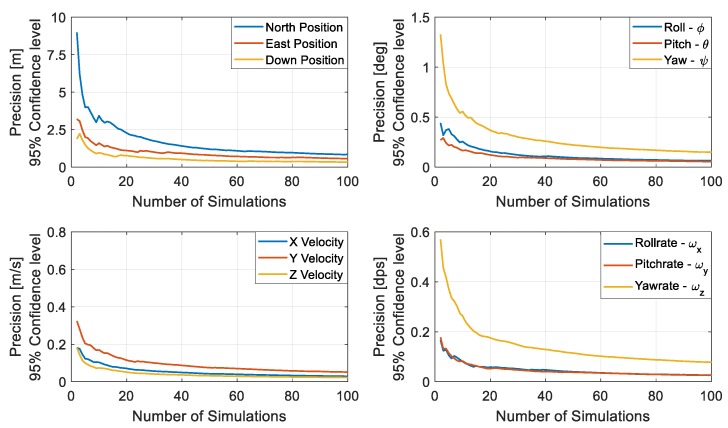
Precision measures for position (**top left**), velocity (**bottom left**), attitude (**top right**), angular rate (**bottom right**) for 95% confidence level for different number of simulations.

A hundred Monte Carlo runs were performed in Matlab to evaluate autonomous navigation performance and investigate the system robustness against random initialisation errors and sensor errors. Evaluating the sample statistics for different runs revealed that 60 Monte Carlo runs resulted in a precision, difference between the population mean and sample mean, of less than 1 metre in all the estimated position states with a 95% confidence level, as can be seen in Figure 7. With 60 runs, the precision for all the other states is well below the uncertainty bounds considered for the states. The combined standard deviation from different sample runs for each state is used in evaluating the precision for the state. Therefore, 100 Monte Carlo runs seemed reasonable in attaining a precision of less than 1 metre in the estimation of position states whilst guaranteeing similarly lower precision for all the other states and include a margin of safety to account for some of the underlying assumptions adopted.

**Figure 8:**
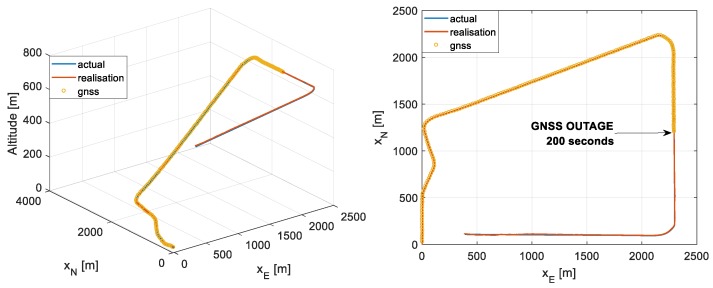
3D flight profile (**left**) and 2D flight profile (**right**).

The same trajectory is used for all of the scenarios and the wind profile is kept constant during the runs. The trajectory is presented in Figure 8, which includes a take-off segment which the autopilot system marks as complete at an altitude of 200 m, a climb segment which completes at 700 m, and a cruise segment where a GNSS outage is induced followed by a descent approach segment. The outage is induced 200 s into the flight and lasts for 140 s. The total flight time is 340 s. The impact of autopilot agility on navigation performance is not investigated in this research.

**Figure 9:**
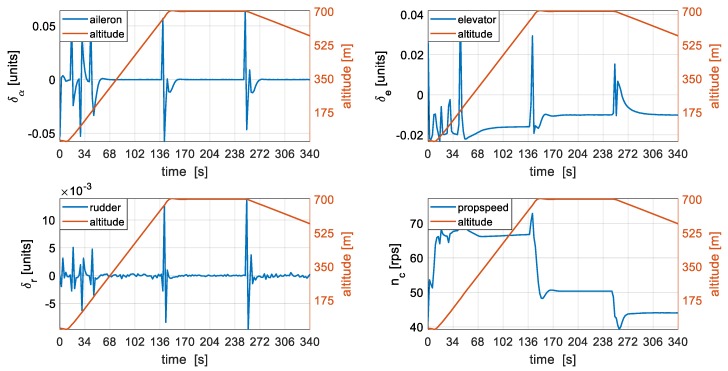
Control activity during the flight.

Control activity during the different flight segments is presented in Figure 9 with altitude used as a representative of the flight segment. The commands from the autopilot are logged at 100 Hz and used alongside simulated sensor data and processed with the developed algorithm.

## 4. Results

**Figure 10:**
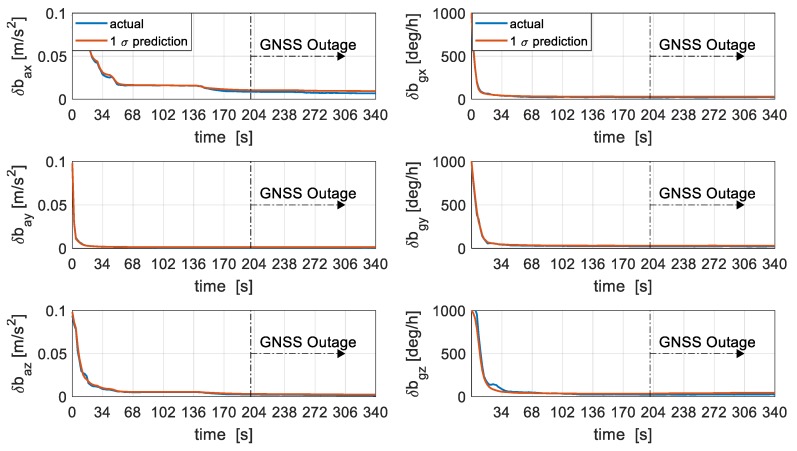
IMU errors estimation, accelerometer errors (**left**) and gyroscope errors (**right**).

The accelerometer and gyroscope bias estimation error is presented in Figure 10. The filter is able to effectively and consistently estimate accelerometer and gyroscope bias errors within the first 50 s with GNSS presence. Ninety-eight percent of the initial turn-on gyroscope bias and bias variation are resolved well within 100 s of GNSS presence. The errors are also seen to be bounded during 140 s of GNSS outage owing to the inherent ability of the dynamics model to offer a bounded navigation solution during periods of GNSS outage. Also, the 1σ prediction error is seen to be consistent with the true error, attributed to a correctness in filter setup.

Results indicate that the *Z*-axis and *X*-axis accelerometer bias are slightly delayed in their estimation, this might be attributed to the inherent coupling between the gyroscope and accelerometer errors under low dynamics and more separation and observability might be achieved with high dynamics.

**Figure 11:**
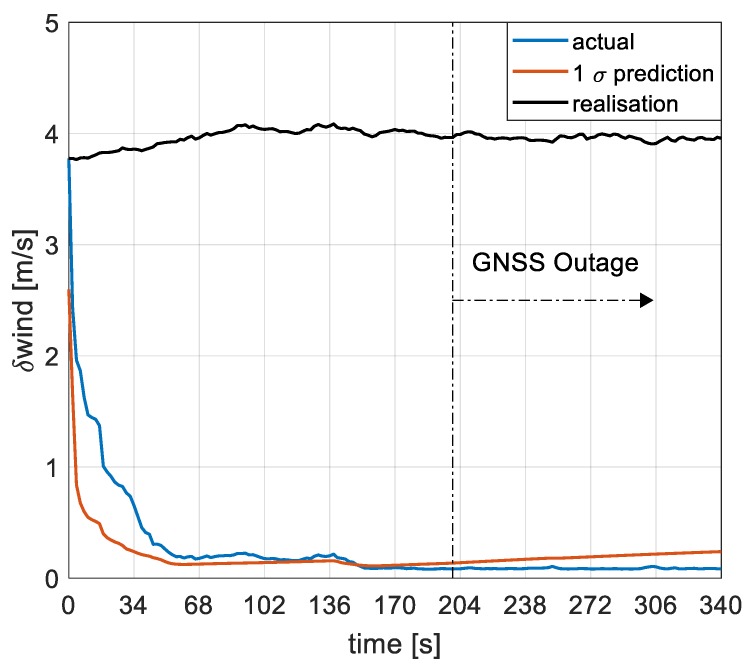
Wind speed errors estimation.

Wind magnitude error is shown in Figure 11. As stated earlier, a slow varying wind is implemented in simulation and the filter estimates the constant component of wind during the flight. Wind is estimated within 60 s of GNSS presence and the error is less than 0.12m/s 150 s into the flight. The error in wind speed estimation remains bounded even after 140 s of GNSS outage, with the final wind estimation error being less than 0.2m/s. The filter is also seen to be consistent in the estimation of wind speed as it can be seen from the 1 σ prediction even without an air data system attributed to correctness in the filter setup and the ability of the UKF to capture higher-order moments. The navigation performance of a model-based approach is generally prone to errors resulting from unknown external disturbances, such as wind, and therefore the filters ability to estimate wind even without an air data system makes this approach robust against external wind disturbances.

**Figure 12:**
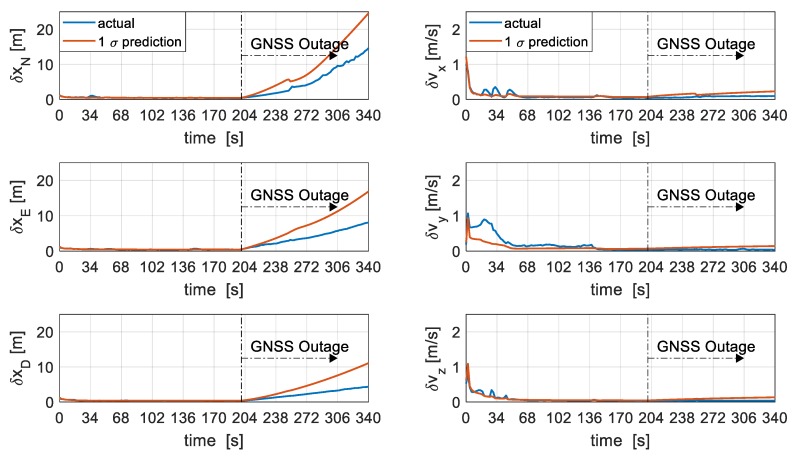
Position (**left**) and velocity (**right**) errors.

The position errors are presented in Figure 12 in the local level frame. It can be seen that for the most part during periods of GNSS availability, the filter prediction is consistent with the actual errors except during short periods of high dynamics between 34 s and 50 s where the filter slightly underestimates the north and east position errors. The slight underestimation might be attributed to the unresolved initialisation errors. During periods of GNSS outage, the filter is seen to be slightly conservative in prediction of position errors, however, the growth rate is rather slow reaching only 14.5 m in the North channel, 8 m in the East, and 4.3 m in the Down direction after 140 s of VDM coasting. The slight conservative prediction of position errors during periods of GNSS outage might be attributed to the coupling between wind speed and position errors.

Velocity is well-estimated within the first 60 s of GNSS availability and the error is seen to be less than 0.1m/s after 150 s of GNSS availability. After resolving initialisation errors, the filter is seen to be consistent in the estimation of velocity in the body-fixed frame. There is a marginal growth in velocity errors after GNSS outage, but errors remain well within 0.1m/s with very slight conservative 1 σ prediction, as can be seen in Figure 12.

**Figure 13:**
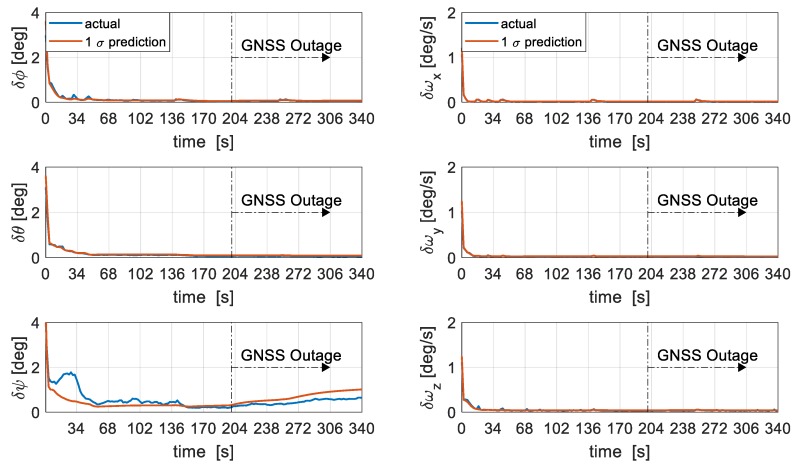
Attitude (**left**) and angular velocity (**right**) errors.

Roll and pitch attitude errors are estimated well within 50 s with GNSS availability but yaw errors are slightly delayed and only well-resolved after 80 s of GNSS availability, as seen in Figure 13. The lack of a direct heading reference as well as large initialisation errors in heading might be the cause of the delayed and slightly optimistic estimate during GNSS availability. The angular rates are quickly estimated within 20 s of GNSS availability due to the presence of direct observations from the gyroscope. Roll and pitch errors remain within 0.06 degrees after 140 s of GNSS outage while yaw error increases slowly and remains within 0.65 degrees after 140 s of GNSS outage. The 1 σ prediction is seen to be consistent with the true error even during periods of GNSS outage.

**Figure 14:**
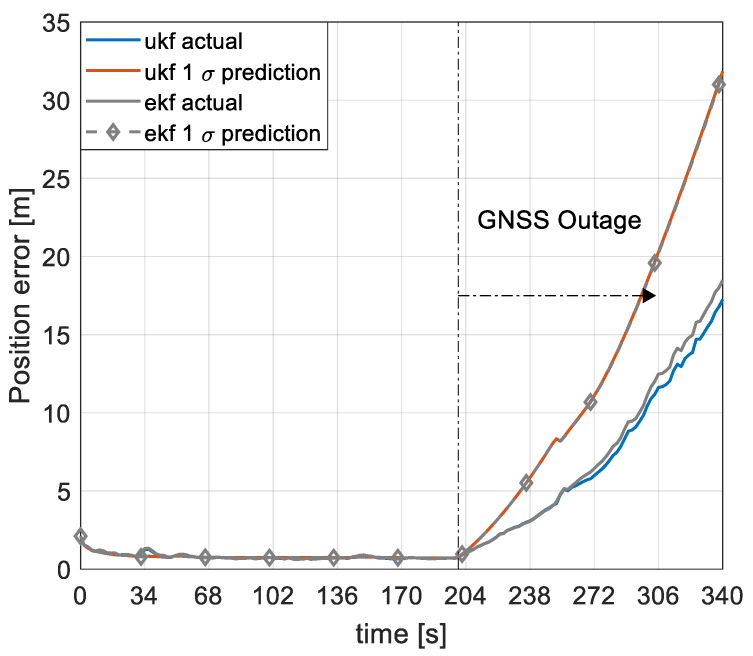
Root mean square (RMS) of position error and 1 σ prediction for the UKF/VDM and EKF/VDM architecture with perturbed moment of inertia terms.

Figure 14 shows the root mean squared (RMS) of position errors for the proposed UKF/VDM architecture with perturbed and augmented moment of inertia terms compared to the EKF/VDM architecture with perturbed but not augmented moment of inertia terms. Two important deductions are made from the results. First, the navigation performance in terms of position errors is similar for the two setups with marginal differences for 100 Monte Carlo runs. Secondly, both setups provide consistent position estimates and the differences are well within the precision of the Monte Carlo runs. It is important to note that these deductions are relatively similar for the remaining navigation states. Even though the relative strength of the proposed architecture as opposed to the EKF/VDM architecture is not obvious at this point, the similarities in navigation performance validates the proposed architecture.

**Figure 15:**
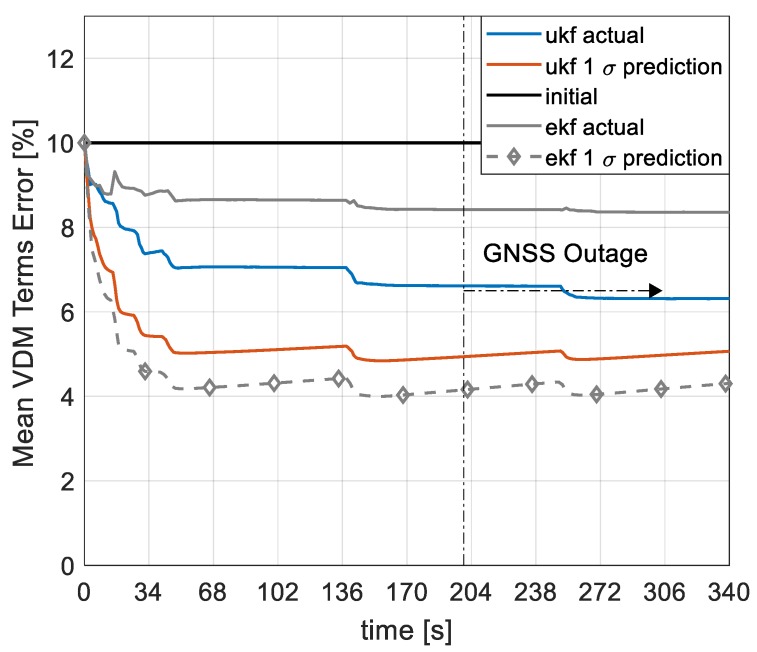
Mean VDM parameters and inertia errors.

The RMS of the mean error of 22 VDM parameters and 4 moment of inertia terms, collectively addressed as VDM terms in this context, is presented in Figure 15. With an initial uncertainty of 10% considered in each of the VDM terms, the filter is seen to be slightly optimistic in their estimation. Generally, the initial error in the VDM terms is seen to reduce quickly during periods of GNSS availability and the mean of the VDM terms remains bounded during periods of GNSS outage. The mean VDM error reduces quickly to less than 7.5% within 50 s of GNSS availability and then gradually to 6.6% at the onset of the GNSS outage. The optimistic filter estimation might be attributed to the strong coupling and correlation of the VDM terms but the difference between the mean error and the filter prediction is less than 19% at the end of the flight. Perhaps, more dynamic manoeuvres, exciting different modes, could improve their overall estimation but the current results are deemed well enough for the purpose of navigation owing to the consistent estimate in navigation states. It is worth mentioning that, for a similar setup, the EKF/VDM architecture with perturbed but not augmented inertia terms is seen to be overly optimistic in the estimation of the VDM parameters with a 48.5% difference between the mean error and the prediction at the end of the flight. This is not very surprising and highlights the strength of the proposed UKF/VDM architecture in the estimation of highly correlated terms. This also shows that the proposed architecture could be used with greater confidence in the presence of inertia tensor perturbations saving both time and cost associated with inertia modelling.

**Figure 16:**
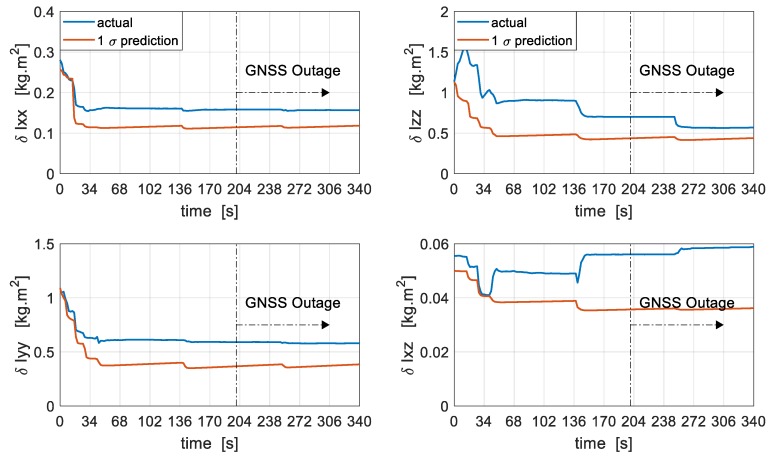
Moment of Inertia calibration. ${\delta I}_{\mathrm{xx}}{,\;\delta I}_{\mathrm{yy}}{,\;\delta I}_{\mathrm{zz}}$ represent the error in the principal inertia terms about the roll, pitch and yaw axis, respectively. ${\delta I}_{\mathrm{xz}}$ represents the error in the product of inertia term.

The error in the estimation of the moment of inertia terms is presented Figure 16. With an initial uncertainty of 10% in the moment of inertia terms it can be seen that errors in roll and pitch inertia terms are quickly resolved within 50 s of GNSS availability, even though the filter appears to be slightly optimistic in estimating these terms. The estimates remain bounded even during periods of extended GNSS outage lasting 140 s and the difference between the filter’s estimate and the actual error at the end of the flight is 24.4% and 33% for roll and pitch inertia terms, respectively. The moment of inertia term in yaw seems to be resolved after the initial errors in the roll and pitch terms have been resolved and continues to be observable even during periods of GNSS outage with a difference of 23% between the filter’s estimate and the actual error at the end of the flight. The product of inertia term seems to be slightly estimated in the initial phase of the flight within 30 s but then gradually diverges afterwards for the remainder of the flight. This might be attributed to the degree of coupling and lack of enough excitation for the product of inertia to be observable and perhaps more dynamic manoeuvres could improve the overall estimation.

Since the filter has shown consistency in the estimation of the navigation states, IMU errors, and wind, whilst being slightly optimistic in the estimation of VDM terms, the degree of correlation between the actual errors and the filter prediction warrants further discussion on uncertainty evolution of all states as well as correlation between the states.

**Figure 17:**
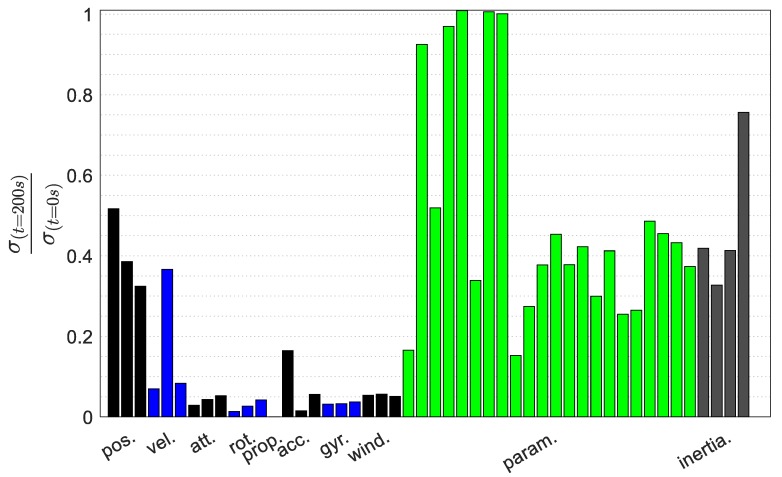
VDM states uncertainty evolution during periods of GNSS availability.

The uncertainty evolution of all the states during periods of GNSS availability is presented in Figure 17. The uncertainty evolution during GNSS availability is presented as a ratio of the uncertainty at the moment of GNSS outage (200 s) to the uncertainty at initialisation. Generally, most states, including the VDM parameters and moments of inertia terms, are observable and remain bounded during GNSS availability. Some parameters including thrust coefficients and lift coefficients are not very well-estimated during periods of GNSS availability. Generally, the observability of VDM parameters is trajectory-dependent but the estimation is sufficient for the purpose of navigation.

**Figure 18:**
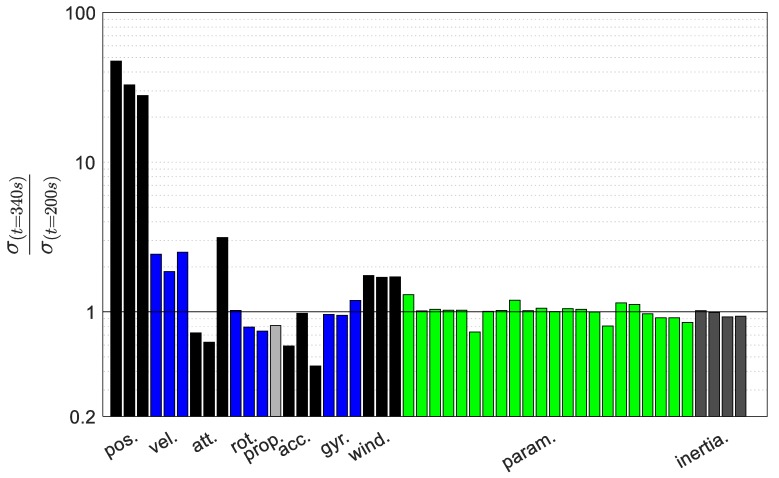
VDM states uncertainty evolution during GNSS outage.

The uncertainty evolution of all of the states during 140 s of GNSS outage is presented in Figure 18. This is presented as a ratio of the uncertainty at the end of the flight or outage period (340 s) to the uncertainty at the onset of the outage (200 s). Interestingly, the moment of inertia terms are well-bounded during the outage period and some terms are even estimated. Further, the VDM parameters are also well-bounded with the uncertainty of some parameters increasing slightly.

**Figure 19:**
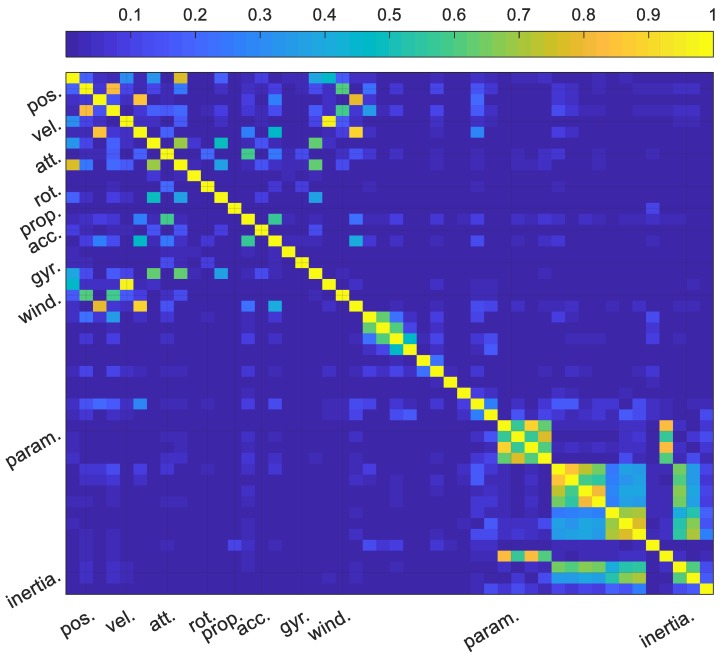
Correlation matrix.

For a better understanding of the correlation properties between the different states, a correlation matrix is presented in Figure 19. It can generally be seen that the moment of inertial terms were decorrelated from most of the navigation states, however, they present strong correlation with the moment derivatives which is not surprising, owing to the formulation of the rigid body equations. Further, it can be seen that the moment derivative terms are correlated within groups. Pitch and yaw moment terms presents significant cross correlation while roll moment terms do not present significant cross correlation with the other terms. The high dynamics in the roll might be the reason for the overall group observability in roll moment terms as opposed to low-level dynamics in pitch and yaw. It’s also important to note that the VDM parameters are well-decorrelated from all the other states. Wind presents significant correlation with position and velocity states and could help explain the significant growth in position error during periods of GNSS outage.

## 5. Conclusions

In this paper, the use of a UKF and direct measurements from a low-cost MEMs-grade IMU and GNSS receiver in a loosely coupled approach has been investigated for a fixed wing UAV. It was found that the approach is able to consistently and efficiently estimate the navigation states whilst also calibrating or estimating model parameters. Further the state vector was augmented to include the moment of inertia terms since it was of interest to estimate this in flight as well. It was shown that the filter was able to estimate the moment of inertia terms even with a 10% initial uncertainty. Further, the filter was able to consistently estimate wind velocity without additional sensors which helped in improving the navigation solution especially during periods of extended GNSS outage where the position error in all directions was shown to be less than 14.5 m. The use of a UKF has been found to produce consistent estimates even with considerable initialisation errors. The real-time implementation of the approach is a subject for future work where other effects could also be investigated including control input noise, presence of coloured noise in measurements, and other practical considerations such as derivation of initial aerodynamic parameters and proper time stamping of measurements and inputs.

## Figures and Tables

**Table 1:** IMU error characteristics.

Property	Accelerometer	Gyroscope
Random bias (σ)	10 mg	1000 °/hr
White noise (PSD)	$100\;\mu g/\sqrt{\mathrm{Hz}}$	21.6°/hr/sqrt(Hz)
First-order Gauss–Markov	0.05 mg	20 °/hr
Correlation Time (τ)	200 s	200 s
Sampling Frequency	100 Hz	100 Hz

**Table 2:** Initial uncertainty [$P_{o}$].

State	Standard Deviation (σ)
Position	1 m
Velocity	[1, 0.5, 0.5] m/s
Attitude	[3.5°, 3.5°, 5°]
Rotation rates	1.5 °/s
Propeller speed	15 rad/s
Model parameters	10%
Moment of Inertia	10%

**Table 3:** Process Noise.

State	Standard Deviation (σ)
Position	$10^{-6}$
Velocity	0.008
Attitude	$10^{-6}$
Rotation rates	$10^{-4}$
Propeller speed Accelerometer Bias Gyroscope Bias Wind	$10^{-4}$ ${2\;\times \;10}^{-5}$ ${2\;\times \;10}^{-6}$ $10^{-3}$
Model parameters	0.015% of True Values
Moment of Inertia	0.015% of True Values

## References

[1] Kim J., Sukkarieh S. A Baro-Altimeter Augmented INS/GPS Navigation System for an Uninhabited Aerial Vehicle. Proceedings of the 6th International Symposium on Satellite Navigation Technology Including Mobile Positioning & Location Services.

[2] George M., Sukkarieh S. Tightly Coupled INS/GPS with Bias Estimation for UAV Applications. Proceedings of the Australasian Conference on Robotics and Automation 2005.

[3] Babu R., Wang J. (2009). Ultra-tight GPS/INS/PL integration: A system concept and performance analysis. GPS Solut..

[4] Brown R., Hwang P.Y. (2012). Introduction to Random Signals and Applied Kalman Filtering.

[5] Falco G., Pini M., Marucco G. (2017). Loose and tight GNSS/INS integrations: Comparison of performance assessed in real Urban scenarios. Sensors.

[6] Hide C. (2003). Integration of GPS and Low Cost INS Measurements. Ph.D. Dissertation.

[7] Wang J., Garratt M., Lambert A., Wang J.J., Han S., Sinclair D. (2008). Integration of Gps/Ins/Vision Sensors to Navigate Unmanned Aerial Vehicles. Int. Arch. Photogramm. Remote Sens. Spat. Inf. Sci..

[8] Lau T.K., Liu Y.H., Lin K.W. (2013). Inertial-based localization for unmanned helicopters against GNSS outage. IEEE Trans. Aerosp. Electron. Syst..

[9] Quinchia A.G., Falco G., Falletti E., Dovis F., Ferrer C. (2013). A comparison between different error modeling of MEMS applied to GPS/INS integrated systems. Sensors.

[10] Papadimitratos P., Jovanovic A. Protection and fundamental vulnerability of GNSS. Proceedings of the 2008 IEEE International Workshop on Satellite and Space Communications.

[11] Tawk Y., Tomé P., Botteron C., Stebler Y., Farine P.-A. (2014). Implementation and Performance of a GPS/INS Tightly Coupled Assisted PLL Architecture Using MEMS Inertial Sensors. Sensors.

[12] Groves P.D. (2008). Principles of GNSS, Inertial, and Multisensor Integrated Navigation Systems.

[13] Madison R., Andrews G., DeBitetto P., Rasmussen S., Bottkol M. Vision-Aided Navigation for Small UAVs in GPS-Challenged Environments. Proceedings of the AIAA Infotech at Aerospace Conference.

[14] Beard R.W., McLain T.W. (2013). Small Unmanned Aircraft: Theory and Practice.

[15] Vasconcelos J.F., Silvestre C., Oliveira P., Guerreiro B. (2010). Embedded UAV model and LASER aiding techniques for inertial navigation systems. Control Eng. Pract..

[16] El-Diasty M., Pagiatakis S. (2009). A Rigorous Temperature-Dependent Stochastic Modelling and Testing for MEMS-Based Inertial Sensor Errors. Sensors.

[17] Koifman M., Bar-Itzhack I.Y. (1999). Inertial navigation system aided by aircraft dynamics. IEEE Trans. Control Syst. Technol..

[18] Bryson M., Sukkarieh S. Vehicle Model Aided Inertial Navigation for a UAV using Low-cost Sensors. Proceedings of the Australasian Conference on Robotics and Automation 2004.

[19] Crocoll P., Görcke L., Trommer G.F., Holzapfel F. (2013). Unified Model Technique for Inertial Navigation. NAVIGATION.

[20] Khaghani M., Skaloud J. (2016). Autonomous Vehicle Dynamic Model-Based Navigation for Small UAVs. NAVIGATION.

[21] Crocoll P., Seibold J., Scholz G., Trommer G.F. (2014). Model-Aided Navigation for a Quadrotor Helicopter: A Novel Navigation System and First Experimental Results. NAVIGATION.

[22] Sendobry A. (2014). Control System Theoretic Approach to Model Based Navigation.

[23] Lyu P., Lai J., Liu J., Zhang L., Liu S. A novel integrated navigation system based on the quadrotor dynamic model. Proceedings of the 2018 IEEE/ION Position, Location and Navigation Symposium (PLANS).

[24] Julier S.J., Durrant-whyte H.F. (2003). On the Role of Process Models in Autonomous Land Vehicle Navigation Systems. IEEE Trans. Robot. Autom..

[25] Vissière D., Bristeau P.-J., Martin A.P., Petit N. Experimental autonomous flight of a small-scaled helicopter using accurate dynamics model and low-cost sensors. Proceedings of the 17th World Congress of the International Federation of Automatic Control.

[26] Dadkhah N., Mettler B., Gebre-egziabher D. A Model-Aided AHRS for Micro Aerial Vehicle Application. Proceedings of the 21st International Technical Meeting of the Satellite Division of The Institute of Navigation (ION GNSS 2008).

[27] Crocoll P., Trommer G.F. Quadrotor Inertial Navigation Aided by a Vehicle Dynamics Model with In-Flight Parameter Estimation. Proceedings of the 27th International Technical Meeting of the Satellite Division of The Institute of Navigation (ION GNSS+ 2014).

[28] Mueller K., Crocoll P., Trommer G.F. Model-Aided Navigation with Wind Estimation for Robust Quadrotor Navigation. Proceedings of the 2016 International Technical Meeting of the Institute of Navigation.

[29] Khaghani M., Skaloud J. (2018). Assessment of VDM-based autonomous navigation of a UAV under operational conditions. Rob. Auton. Syst..

[30] Sendobry A. A Model Based Navigation Architecture for Small Unmanned Aerial Vehicles. Proceedings of the European Navigation Conference.

[31] Khaghani M., Skaloud J. VDM-based UAV Attitude Determination in Absence of IMU Data. Proceedings of the European Navigation Conference, ENC 2018.

[32] Zahran S., Moussa A., El-Sheimy N., Sesay A.B. (2018). Hybrid Machine Learning VDM for UAVs in GNSS-denied Environment. NAVIGATION.

[33] Mohammadkarimi H., Nobahari H. (2018). A Model Aided Inertial Navigation System for Automatic Landing of Unmanned Aerial Vehicles. NAVIGATION.

[34] International Civil Aviation Organization (1993). Manual of the ICAO Standard Atmosphere Extended to 80 Kilometres (262,500 Feet).

[35] Ducard G. (2007). Fault-Tolerant Flight Control and Guidance Systems for a Small Unmanned Aerial Vehicle.

[36] Julier S.J., Uhlmann J.K. (1997). New extension of the Kalman filter to nonlinear systems. Signal Process. Sensor Fusion Target Recogn..

[37] Julier S.J., Uhlmann J.K., Durrant-whyte H.F. A New Approach for Filtering Nonlinear Systems. Proceedings of the American Control Conference.

[38] Gelb A., Kasper J.F., Nash R.A., Price C.F., Sutherland A.A. (1974). Applied Optimal Estimation.

